# Time-dependent regulation of morphological changes and cartilage differentiation markers in the mouse pubic symphysis during pregnancy and postpartum recovery

**DOI:** 10.1371/journal.pone.0195304

**Published:** 2018-04-05

**Authors:** Bianca Gazieri Castelucci, Sílvio Roberto Consonni, Viviane Souza Rosa, Lucimara Aparecida Sensiate, Paula Cristina Rugno Delatti, Lúcia Elvira Alvares, Paulo Pinto Joazeiro

**Affiliations:** Department of Biochemistry and Tissue Biology, Institute of Biology, State University of Campinas (UNICAMP), Campinas, Brazil; University of Umeå, SWEDEN

## Abstract

Animal models commonly serve as a bridge between *in vitro* experiments and clinical applications; however, few physiological processes in adult animals are sufficient to serve as proof-of-concept models for cartilage regeneration. Intriguingly, some rodents, such as young adult mice, undergo physiological connective tissue modifications to birth canal elements such as the pubic symphysis during pregnancy; therefore, we investigated whether the differential expression of cartilage differentiation markers is associated with cartilaginous tissue morphological modifications during these changes. Our results showed that osteochondral progenitor cells expressing *Runx2*, *Sox9*, *Col2a1* and *Dcx* at the non-pregnant pubic symphysis proliferated and differentiated throughout pregnancy, giving rise to a complex osteoligamentous junction that attached the interpubic ligament to the pubic bones until labour occurred. After delivery, the recovery of pubic symphysis cartilaginous tissues was improved by the time-dependent expression of these chondrocytic lineage markers at the osteoligamentous junction. This process potentially recapitulates embryologic chondrocytic differentiation to successfully recover hyaline cartilaginous pads at 10 days postpartum. Therefore, we propose that this physiological phenomenon represents a proof-of-concept model for investigating the mechanisms involved in cartilage restoration in adult animals.

## Introduction

Worldwide, many people suffer from problems in bodily cartilaginous structures caused by injuries, degenerative diseases or ageing. To restore articular tissue functions, researchers have sought to develop therapeutic approaches using cells, biomaterial matrices and tissue-engineered grafts to recapitulate events of pre-cartilaginous mesenchymal condensation and to stimulate cartilage regeneration and turnover [1–3]. Preclinical studies in animal models, such as mice and rabbits, are essential for the development of new therapeutic strategies, serving as a bridge between *in vitro* experiments and clinical tests in humans [4, 5]. Despite the intrinsic healing potential of rodent cartilaginous structures, few physiological processes represent a proof-of-concept model for the investigation of cartilage regeneration in adult animals [4, 5].

Studies with rodents have revealed important physiological connective tissue modifications of birth canal elements during pregnancy in young adults [6–9]. In these animals, the pubic symphysis (PS), an amphiarthrodial joint between the pubic bones, undergoes drastic remodelling, thereby allowing the passage of offspring during labour and, after delivery, restructuring of the pelvic girdle, thus restoring pelvic floor homeostasis [10–15]. During pregnancy, a process primarily induced by relaxin and oestrogen promotes interpubic ligament (IpL) formation, which replaces the PS. IpL development leads to pubic bone separation and enlargement of the interpubic gap, which is necessary for delivery [6, 12, 16–21]. Mouse PS remodelling arises from alterations in the extracellular matrix (ECM) composition and histoarchitecture under the influence of matrix metalloproteinases (MMPs), tissue inhibitors of metalloproteinases (TIMPs) and inducible nitric oxide synthase (iNOS) activity in the interpubic tissues [22–24]. These modifications include changes in collagen and elastic fibre alignment and solubility [12, 15, 25], as well as the proteoglycan and hyaluronic acid composition of the PS [26–28]. PS histoarchitecture recovery occurs between 10 and 40 days postpartum (dpp), thereby resulting in the restoration of its function to support pelvic organs and dissipate local mechanical forces [15, 29]. This phenomenon, previously termed “PS metamorphosis” [30], involves the rapid turnover of both cartilaginous and bone tissue. Cartilaginous cells in the PS have elongated or angular shape phenotypes and are believed to coordinate this joint remodelling during pregnancy and postpartum [10, 11]. These osteochondral progenitor-like cells and chondrocytes located in the PS have a well-established capacity to respond to relaxin and oestrogen, hormones that affect chondrocyte differentiation and gene expression [16, 31–33].

During embryonic development, *Indian hedgehog (Ihh)*, *Sonic hedgehog* (*Shh*) and *Wingless-related integration site (Wnt)* signalling pathways regulate PS and pelvic girdle formation [34]. These pathways modulate the activity of *bone morphogenetic proteins* (*BMPs)*, *Noggin* and other key factors that are responsible for osteochondrogenesis [35]. Together, these signalling molecules are crucial regulators of cartilage and bone formation during embryogenesis and postnatal life and activate specific transcription factors in progenitor cells [34–38]. In particular, *SRY-Related High-Mobility Group Box 9* (*Sox9*) and *Runt-related transcription factor 2* (*Runx2*) are necessary for osteochondral progenitor cell differentiation. These factors activate the synthesis of cartilage and bone ECM components, such as type I and II collagen, hyaluronic acid and aggrecan. In adults, *Sox9* and *Runx2* expression at endochondral growth plates drive chondrocyte differentiation and maturation, thereby maintaining articular cartilage organization. In addition, these factors control transcription levels of *doublecortin* (*Dcx)* and *growth/differentiation factor 5 (Gdf5)*, proteins that play key roles in joint development [37, 39–43].

Given the importance of shedding light on the mechanisms involved in the formation and repair of connective tissue for human health, we used the remodelling of mouse PS as a model to investigate these processes. Classical studies have evaluated the responsiveness of osteoprogenitor-like cells to oestrogen [31] and characterized metachromasia and tissue morphology [10, 11, 16, 44]. However, few studies have focused on the association between morphology and the expression of cartilage differentiation markers during PS remodelling. Therefore, this work presents a detailed analysis of *Runx2*, *Sox9*, *type II collagen (Col2a1)* and *Dcx* expression at the interface between the pubic bones and PS cartilage as well as the drastic tissue remodelling and recovery after first pregnancy in non-pregnant (NP), pregnant and postpartum female mice. Our results revealed that progenitor cells expressing these markers at NP PS proliferate and differentiate throughout pregnancy to give rise to a complex osteoligamentous junction that attaches IpL to pubic bones until labour occurs. After delivery, the progressive recovery of interpubic joint histoarchitecture involves a time-dependent expression of cartilage markers at the osteoligamentous junction until the complete restoration of PS hyaline cartilage at 10dpp. Therefore, the dynamic behaviour of cells and tissues during the different phases of PS remodelling are associated with specific patterns of expression. These findings motivate us to propose that the physiological phenomenon characterized in our study represents a proof-of-concept model for investigating cartilage restoration in adult animals.

## Results

### PS remodelling during pregnancy and postpartum

To characterize PS histoarchitectural changes during mouse pregnancy and postpartum, we performed a systematic analysis of tissue morphology. This was done using standardized histoprocessing techniques and an unbiased evaluation of tissue quality by choosing only plastic sections with no artefactual tears, holes or folds for analysis.

**Fig 1:**
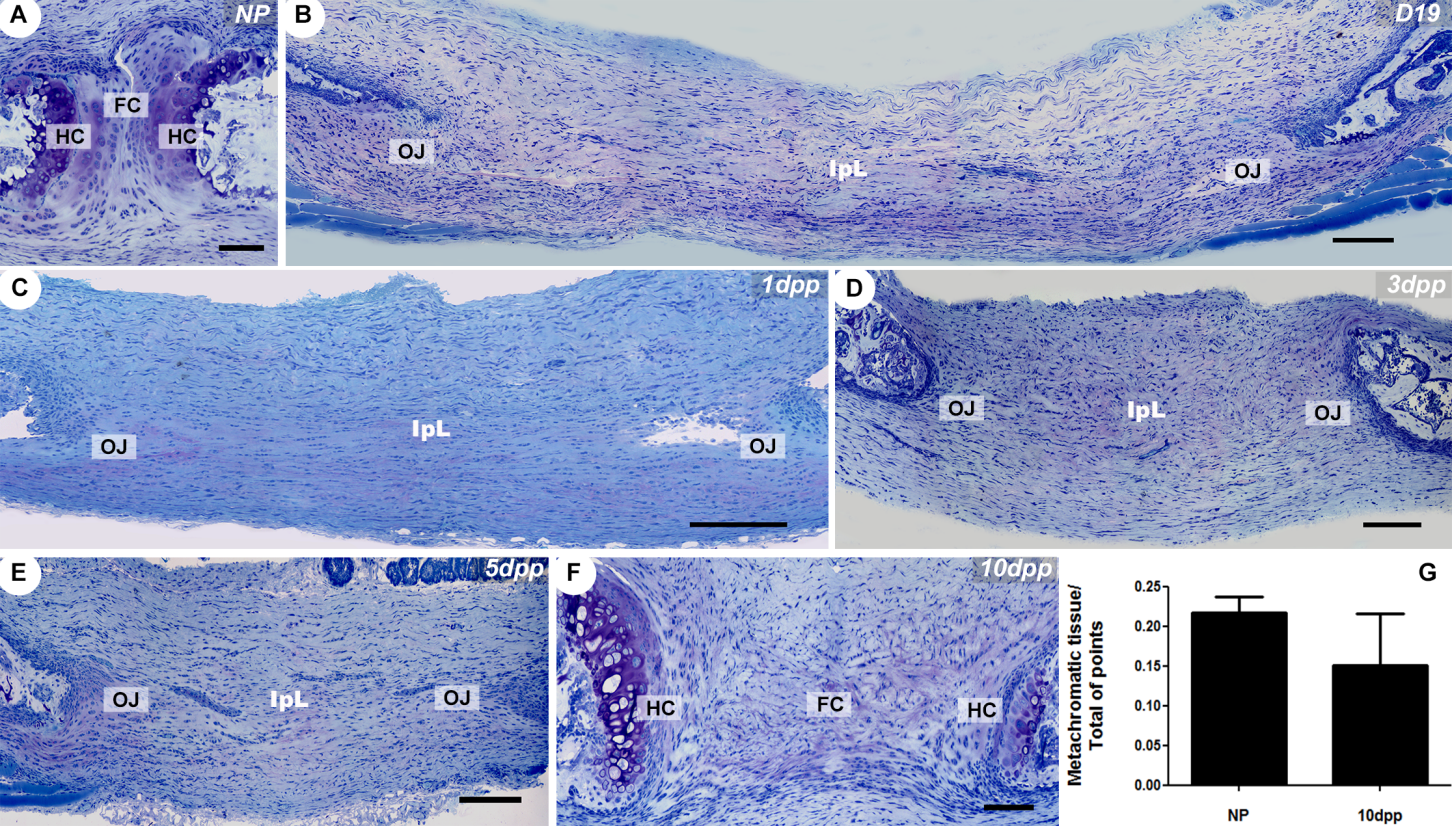
Dynamic changes in the histoarchitecture and ECM deposition accompany PS remodelling. (A-F) Giemsa staining of PS and IpL transverse sections. (A) NP PS consists of a narrow fibrocartilaginous disc (FC) situated between two high metachromatic hyaline cartilaginous pads (HC) on the surface of the subchondral pubic bones, which are caudally and ventrally connected by non-metachromatic dense connective tissue. (B-E) The absence of highly metachromatic hyaline cartilage (HC) and the presence of IpL attached to the pubic bone via an osteoligamentous junction (OJ). (F) The return of similar NP PS histoarchitecture and highly metachromatic hyaline cartilage (HC) tissue at 10dpp in the PS. (G) Morphometric measurement of hyaline cartilage metachromatic tissues volume in the NP and 10dpp mice PS (U = 3; p = 0.7). Data from a Mann-Whitney test are presented with the means and SEM. (A, F) Scale bars = 50 μm. (B-E) Scale bars = 100 μm.

Our results showed that during pregnancy, a fibrocartilaginous disc sandwiched between highly metachromatic hyaline cartilaginous pads in the NP PS gives rise to the interpubic ligament, which is attached to the pubic bones via the osteoligamentous junction (Fig 1A). From 19 days of pregnancy (D19) until 1dpp, there is a decrease in mucopolysaccharide ECM deposition at the osteoligamentous junction as revealed by low or absent metachromatic staining of samples. The ECM composition of interpubic gap cartilaginous tissues starts to be restored approximately 5dpp, when faded metachromatic staining is observed in the osteoligamentous junction fibrocartilage. The postpartum remodelling also leads to IpL reabsorption and to hyaline cartilage histoarchitecture and ECM mucopolysaccharide composition recovery at 10dpp, as revealed by the return of highly metachromatic staining at this structure (Fig 1A–1F). Metachromatic tissue volumes in NP and 10dpp hyaline cartilage pads were morphometrically estimated by point counting, and the numerical data demonstrated no significant differences between the groups (Fig 1G).

**Fig 2:**
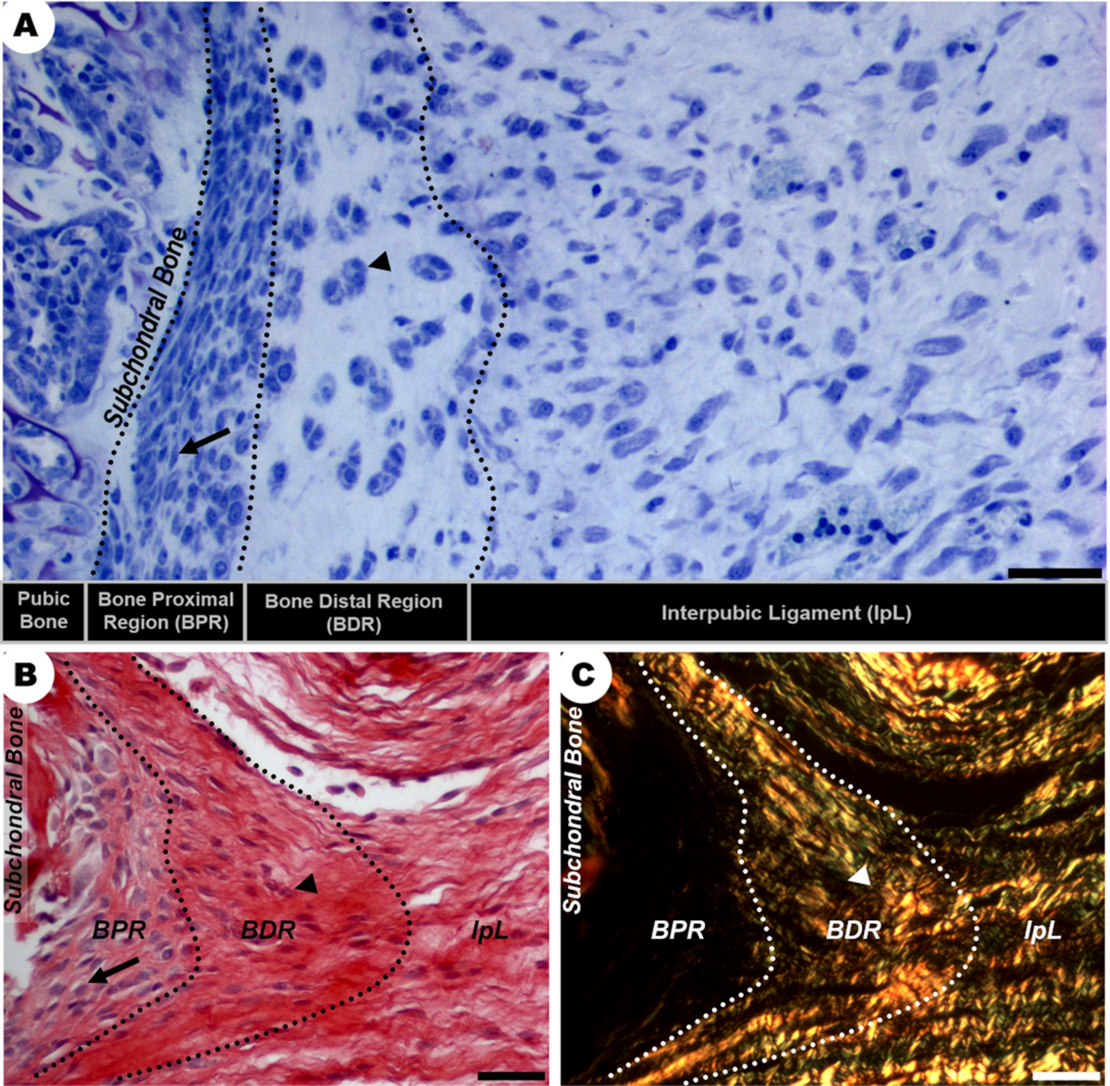
Morphological characterization of distinct regions of the IpL osteoligamentous junction at D19. (A-C) There are two distinct regions in the IpL osteoligamentous junction (dotted line): the BPR is composed of elongated cells (arrows) with poorly birefringent collagen fibrils at the ECM, while the BDR contains angular chondrocyte-like cells (arrowheads) organized as isogenous groups surrounded by dense bundles of collagen. (A) Giemsa staining, scale bar = 30 μm. (B, C) Sirius Red staining and polarized microscopy, scale bar = 20 μm.

From D19 to 5dpp, the IpL osteoligamentous junction present at the interpubic gap comprised two major regions: the bone proximal region (BPR) next to the subchondral bone surface and the bone distal region (BDR) interconnected with the IpL (Fig 2A). While the BPR was a perichondrium like-tissue composed of a highly cellularized mass of elongated mesenchymal-like cells surrounded by a slightly acidophilic and birefringent matrix, the BDR was a region of angular chondrocyte-like cells supported by a birefringent and acidophilic collagen network, which was continuous to the IpL (Fig 2B and 2C).

**Fig 3:**
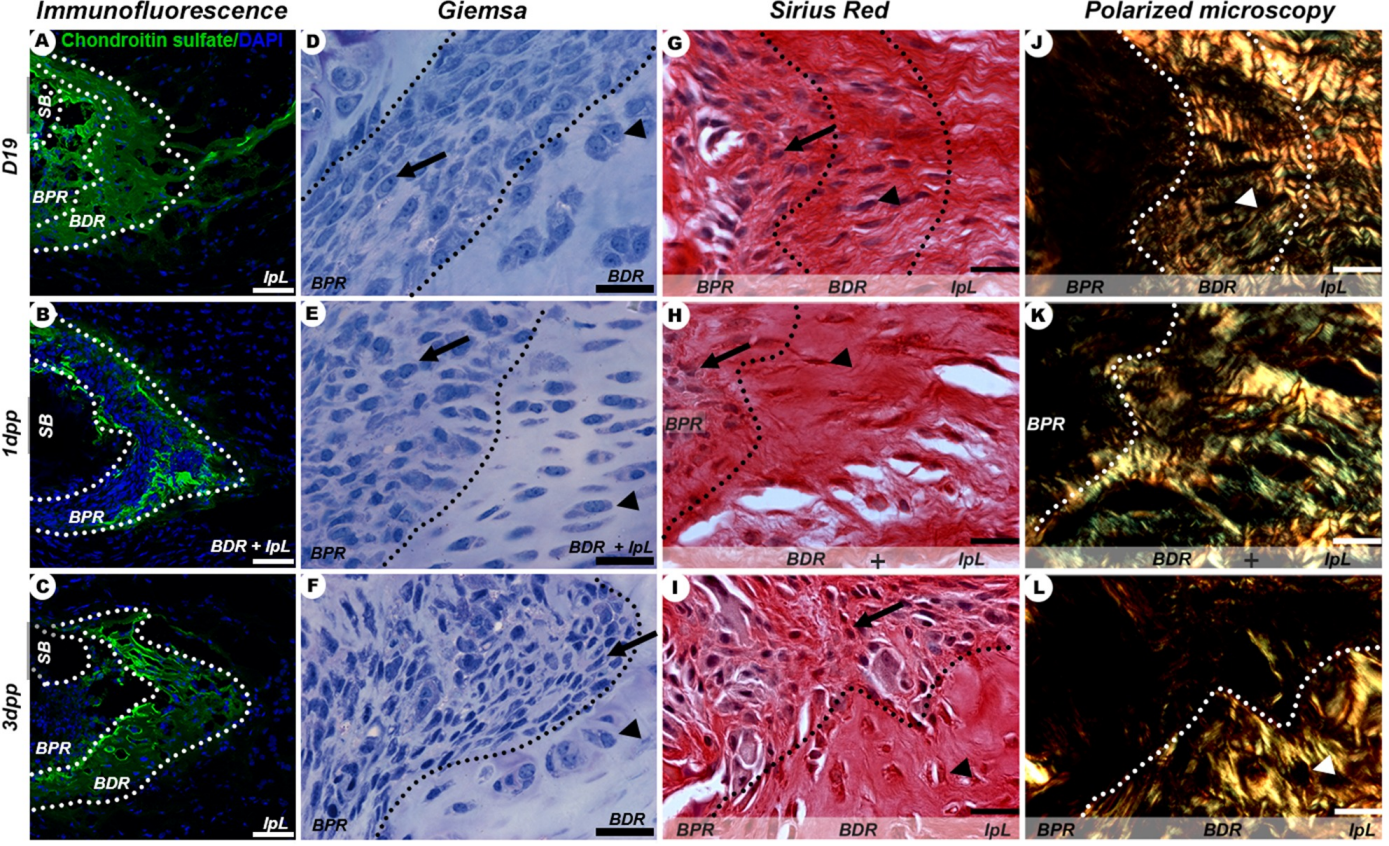
IpL osteoligamentous junction remodelling during late pregnancy (D19) and early postpartum (1-3dpp). (A-C) Identification of chondroitin sulphate at BDR and BPR regions (dotted lines). (D-I) Typical cell phenotypes present in BPR (arrow) and BDR (arrowhead). (J-L) Isogenous groups of chondrocytes seen as dark areas (arrowhead) distributed in BDR and BPR regions that exhibit differently birefringent collagenous ECM. Scale bar = 20 μm.

Qualitatively, there was a progressive increase in BPR development at the IpL osteoligamentous junction from D19 to 1dpp, while the area of the BDR was gradually reduced (Fig 3A and 3B). From D19 to 1dpp, BDR chondroitin sulphate-rich ECM was reabsorbed and its cellular composition changed from angular chondrocyte-like cells to elongated cells. During this period, BPR was well developed and consisted of a mass of elongated mesenchymal-like cells immersed in a poorly birefringent ECM near subchondral bone (Fig 3A, 3B, 3D, 3E, 3G and 3H). However, only at 1dpp, BPR had a low chondroitin sulphate content and seemed to be directly attached to the IpL (Fig 3B, 3E, 3H and 3K).

After 3dpp, chondroitin sulphate immunofluorescence and light microscopy indicated a gradual intensification of cartilaginous ECM deposition (Fig 3C, 3F and 3I). At this point, BDR is composed of a compact, highly birefringent and chondroitin sulphate-rich ECM where angular chondrocyte-like cells organized in isogenous groups can be observed (Fig 3C, 3F, 3I and 3L). This chondrocyte differentiation was evidenced in BDR at 3dpp and might contribute to cartilaginous pad restoration.

### Immunolocalization and mRNA expression of cartilage and bone differentiation markers

**Fig 4:**
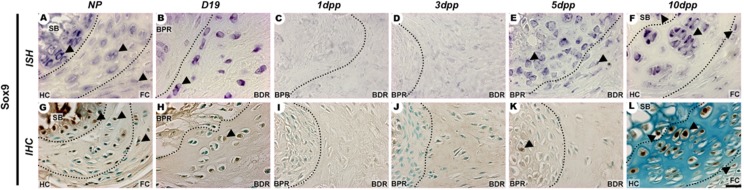
Spatiotemporal expression of Sox9 in the PS cartilage pads and IpL osteoligamentous junction during pregnancy and the postpartum period. (A, F, G and L) Both Sox9 mRNA and protein were localized at the hyaline cartilage (HC) and fibrocartilage (FC) in angular and elliptical chondrocyte-like cells and at round cells near the subchondral bone (SB) in NP and 10dpp PS (arrowheads). (B and H) At the IpL osteoligamentous junction, Sox9 mRNA and protein were observed in elongated cells at the BPR and mainly in BDR angular chondrocyte-like cells at D19 (arrowheads). (E and K) At 5dpp, Sox9 mRNA and protein were localized in round cells at the BPR region of the IpL osteoligamentous junction, but only cells testing positive for *Sox9* mRNA were observed at the BDR (arrowheads). (C, D, I and J) From 1dpp to 3dpp, no cells positive for either Sox9 mRNA or protein were observed at the IpL osteoligamentous junction (1:2000 anti-DIG pod/H-O). *In situ* hybridization (ISH) and Immunohistochemistry (IHC) experiments. Scale bars = 20 μm.

**Fig 5:**
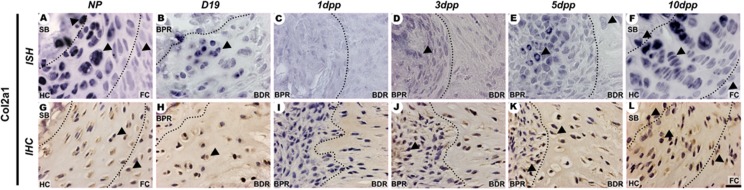
Spatiotemporal expression of Col2a1 in the PS cartilage pads and IpL osteoligamentous junction during pregnancy and the postpartum period. (A- F) Distribution of *Col2a1* mRNA expression in hyaline cartilage (HC), near the subchondral bone (SB), in fibrocartilage (FC) at NP and 1dpp PS, and in BDR and BPR regions of the osteoligamentous junction from D19 to 5dpp. *Col2a1*-positive cells exhibit variable phenotypes: round cells at SB, elongated cells at the BPR, angular chondrocyte-like cells (HC/BDR) and elliptical chondrocyte-like cells (FC) from NP to 10dpp PS (arrowheads). (G-L) Areas temporally immunostaining to procollagen encoded by Col2a1. Positive cells presented a coincident phenotype and localization with *Col2a1* mRNA-positive cells (arrowheads) (1:2000 anti-DIG pod/H-O). *In situ* hybridization (ISH) and Immunohistochemistry (IHC) experiments. Scale bars = 20 μm.

**Fig 6:**
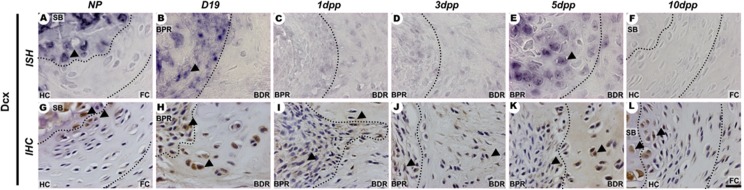
Spatiotemporal expression of Dcx in the PS cartilage pads and IpL osteoligamentous junction during pregnancy and the postpartum period. (A and G) Both Dcx mRNA and protein were observed in round cells near the NP PS subchondral bone (SB) and in hyaline cartilage (HC) angular chondrocyte-like cells (arrowheads). (B, E, H and K). At the osteoligamentous junction, Dcx mRNA and protein were observed in elongated cells and round cells of the BPR region at D19 and 5dpp, respectively (arrowheads). (C, D, F, I, J and L) Only DCX protein-positive cells were localized in both the BPR and BDR regions of the IpL osteoligamentous junction at 1dpp and 3dpp, and this occurred mostly in round cells near the PS subchondral bone (SB) at 10dpp PS (arrowheads). (1:2000 anti-DIG pod/H-O *In situ* hybridization (ISH) and Immunohistochemistry (IHC) experiments. Scale bars = 20 μm.

Sox9 and Col2a1 mRNA expression and protein showed a spatiotemporally specific pattern (Figs 4 and 5) that was related to the increase in metachromatic tissue during PS remodelling (Fig 1G). In the NP PS, Sox9, Col2a1 and Dcx were localized in cells at the hyaline cartilage and close to the subchondral bone (Figs 4A and 4G; 5A and 5G; 6A and 6G). During late pregnancy, *Dcx* mRNA was restricted to cells in the BPR region of the IpL osteoligamentous junction, but cells staining positive for DCX protein were found along all IpL osteoligamentous junction regions. In contrast, cells positive for Sox9 or Col2a1 mRNA and protein were localized mostly in the BDR region of the IpL osteoligamentous junction (Figs 4B and 4H; 5B and 5H; 6B and 6H). Immediately following delivery, there was a notable decrease in mRNA expression and protein localization in cells at the IpL osteoligamentous junction for all those markers (Figs 4C, 4D, 4I and 4J; 5C, 5D, 5I and 5J; 6C, 6D, 6I and 6J). After 5dpp, cells testing positive for *Sox9*, *Col2a1* or *Dcx* mRNA were mainly observed in the BPR region of the IpL osteoligamentous junction, while cells testing positive for COL2A1 or DCX protein were mostly located in the BDR region (Figs 4E and 4K; 5E and 5K; 6E and 6K). However, unlike the progressive increase in Sox9 or Col2a1-positive cells during PS hyaline cartilage formation at 10dpp, there was a clear decrease in *Dcx* mRNA transcription in both the PS hyaline cartilage and fibrocartilage cells (Figs 4F and 4L; 5F and 5L; 6F and 6L).

**Fig 7:**
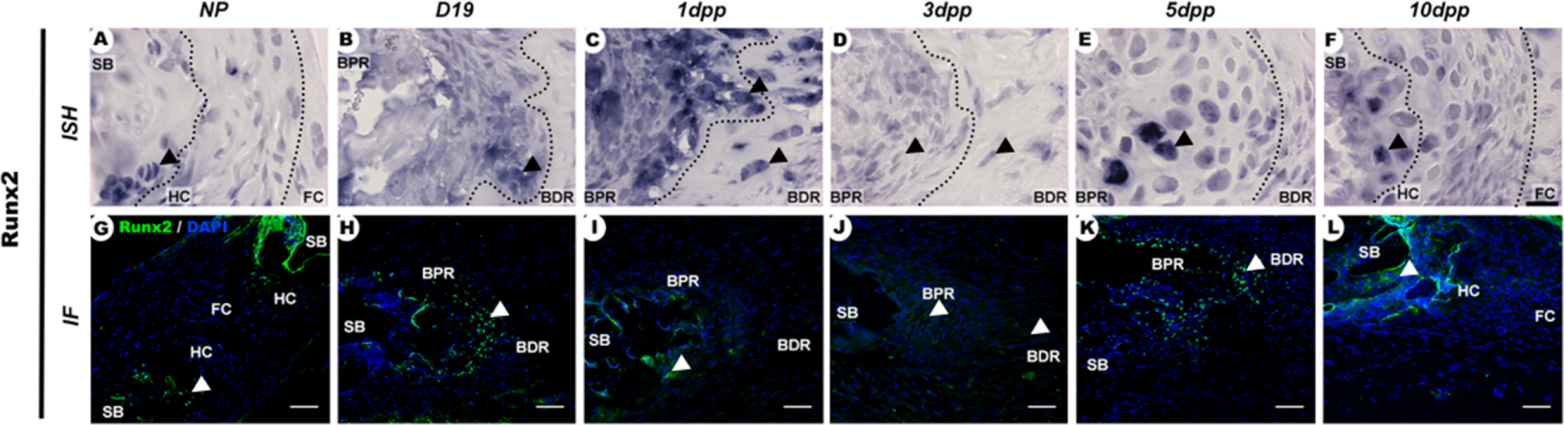
Spatiotemporal expression of Runx2 in the PS cartilage pads and IpL osteoligamentous junction during pregnancy and the postpartum period. (A, F, G and L) Runx2 mRNA and protein were localized in round cells at the subchondral bone (SB) and hyaline cartilage (HC) cells with faded staining in NP and 10dpp PS (arrowheads). (B, C, E, H, I and K) At the IpL osteoligamentous junction, Runx2 mRNA expression and protein were observed primarily in elongated or round cells at the BPR region at D19, 1dpp and 5dpp (arrowheads), (D and J) while at 3dpp, faded staining for mRNA and protein were observed in elongated cells of both the BPR and BDR (arrowheads) regions (1:2000 anti-DIG pod/H-O). *In situ* hybridization (ISH) and Immunohistochemistry (IHC) experiments. (A-F) Scale bars = 20 μm. (G-L) Scale bars = 30 μm.

In NP mice, Runx2 mRNA expression and protein localization were observed in a small group of cells at the subchondral bone (Fig 7A and 7G). From the end of pregnancy up to 1dpp, there was an increase in Runx2 gene expression and protein localization in cells of both the BPR and BDR regions of the IpL osteoligamentous junction (Fig 7B, 7C, 7H and 7I). At 3dpp, Runx2 expression showed a marked decrease in cells of the IpL osteoligamentous junction (Fig 7D and 7J), while both Runx2 mRNA expression and protein localization were increased primarily in cells of the BPR after 5dpp (Fig 7E and 7K). At 10dpp, a small and specific group of round cells at the subchondral bone exhibited both Runx2 mRNA and protein expression (Fig 7F and 7L).

Embryonic cartilaginous blastemal tissue was a consistent positive control for the ISH and immunolocalization assays (S1 Fig).

## Discussion

The qualitative light microscopy analysis presented here describes the morphological and metachromatic tissue remodelling of mouse PS, and the results are consistent with those previously reported by Crelin and Hall [11, 16]. In addition, based on our semi-quantitative morphometric analyses of tissue metachromatic volume, the mucopolysaccharide content of PS hyaline cartilage is restored to NP levels at 10dpp. The cell phenotypes that we observed during our morphological analysis of the BPR and BDR regions of the IpL osteoligamentous junction from late pregnancy to early postpartum were similar in shape and location to those described by Hall [16]. This author reported that the elongated cells located next to the pubic bones during pregnancy and postpartum were similar to mesenchymal cells in shape and organization and that angular or round-shaped chondrocytes could be observed at interpubic joint cartilaginous tissues during PS remodelling. Our histological and chondroitin sulphate immunostaining observations allowed us to describe in detail the morphological aspects of the complex osteoligamentous junction formed in the IpL at the end of pregnancy and its remodelling during the early postpartum period. At late pregnancy, this structure is similar to a fibrocartilaginous *enthesis*, a tendon-to-bone insertion with typical zones that develop during morphogenesis and are characterized by specific cell phenotypes and matrix components [36, 37, 45, 46]. After delivery, gradual tissue modifications lead to cartilage reabsorption, which permits a direct interaction between the osteoligamentous junction BPR and the IpL. This structure is similar to a fibrous *enthesis*, where dense fibrous connective tissue connects the tendon/ligament to the periosteum, and there is no evidence of (fibro)cartilage differentiation [45]. Prior to the complete recovery of hyaline cartilaginous pads at 10dpp, our chondroitin sulphate immunofluorescence results demonstrated that the fibrocartilaginous BDR of the IpL osteoligamentous junction is restored, returning to the similar fibrocartilaginous *enthesis* histoarchitecture that is essential to support the propagation of biomechanical forces through bone attachment sites without inducing breakage or damage under mechanical load [36, 45, 46].

Next to the pubic bones of NP PS mice, we observed the presence of small groups of round cells expressing *Sox9*, *Col2a1*, *Dcx* and *Runx2*, which are similarly expressed by progenitor cells during the early stages of endochondral bone development in the limbs of mouse embryos [39, 43, 47 and 48]. Interestingly, osteochondral progenitor cells at PS during embryological development are sensible to *Hedgehog* signalling in such a way that ectopically induced *Hh* signalling can lead to the onset of omphalocele and pubic diastasis formation [34]. Recently, a broad expression of the BMP antagonists *Noggin* and *Gremlin1* was reported in adult PS cells, indicating that this expression may contribute to the maintenance of appropriate levels of BMP activity for normal cartilage maintenance [35]. Once BMP directly regulates the expression of several chondrocyte-specific genes [49], the presence of *Noggin* and *Gremlin1* BMP regulators in NP PS can also interfere directly with the fate of the pool of round cells that test positive for *Sox9*, *Col2a1*, *Dcx* and *Runx2*, promoting their differentiation into chondroblasts or osteoblasts.

Alterations in the expression of *Sox9*, *Col2a1*, *Dcx* and *Runx2* in round or elongated cells located close the subchondral bones in PS remodelling may be indicative of a pool of osteochondral progenitor cells in a transient state that, eventually, could commit to one or more cell phenotypes depending on the molecular stimuli that they receive, as with the stem cells present in joint subchondral bone and cartilage niches (among others) as reviewed by McGonagle and coworkers [50]. As key regulators of the major pathways involved, *Sox9* and *Runx2* are essential for chondrogenesis and osteogenesis, respectively, and are expressed by a unique progenitor cell population that can express moderate levels of both *Sox9* and *Runx2* that migrate from the bone marrow and support the repair of human articular cartilage during the late stages of osteoarthritis [51].

The continuous presence of *Sox9-* and *Col2a1*-positive cells in the PS fibrocartilage in NP and 10dpp mice, in angular chondrocyte-like cells in D19 mice at the BDR of the IpL osteoligamentous junction and in round cells at the BPR of the IpL osteoligamentous junction after 5dpp suggests that compressive forces act constantly in this region. Similar to *in vitro* mesenchymal cells in 3D culture, in vivo *enthesis* development after birth and to the repair of bone fractures [46, 52, 53, 54], under compressive conditions, histological evaluation of the osteoligamentous junction and PS cell phenotypes and the localization of cartilage markers showed an apparent acceleration in the rate and extent of chondrogenesis. Conversely, at 1dpp, there were decreases in the numbers of cells testing positive for *Sox9* or *Col2a1* and an increase in *Runx2* production by elongated cells at both IpL osteoligamentous regions when compressive forces were disturbed by changes in collagen fibre compaction and hyaluronic acid [27, 28] and versican content [28] and the activity of MMPs, TIMPs and cathepsins [22, 24]. Similar to results described during embryonic mouse endochondral ossification [40], these changes in *Sox9* and *Runx2* expression pattern at 1dpp in IpL osteoligamentous cells can also indicate that *Sox9* is dominant over *Runx2* function in terms of osteochondral progenitors that are destined for a chondrogenic lineage during PS remodelling. Thus, constant compressive loads during PS remodelling appear to favour both the commitment of osteochondral progenitors at the subchondral bone in the PS and BPR regions of osteoligamentous IpL to the chondrocytic lineage and their proliferation and differentiation to mature chondrocytes in hyaline or fibrocartilaginous regions of the interpubic joint during late pregnancy, mostly after 5dpp.

Notably, the expression of *Sox9* and *Col2a1* by chondrocytic lineage cells can also be regulated by hormonal levels. Among the female hormones, oestrogens are important endocrine regulators of skeletal growth and maintenance and can accelerate growth plate fusion by advancing the senescence of the growth plate via the proliferative exhaustion of chondrocytes [55]. Interestingly, according to Wang and coworkers [33], PS cells exhibited significantly higher gene expression levels of the hormonal receptors ER-α (>12 and >4.3-fold), ER-β (>3 and >1.4-fold), LGR7 (>5 and >1.7-fold) and PR (>2.5 and >1.4-fold) than did other joint cells, such as knee meniscus and temporomandibular fibrochondrocytes, making them more responsive to any hormonal variations. In addition, it was previously described that oestrogen, progesterone and relaxin levels are relatively low in mouse serum during the postpartum period compared with the end of pregnancy [9, 56, 57]. Low levels of oestrogen permit the interaction of factors that bind to the *Col2a1* and *Sox9* promoters and enhance their expression in undifferentiated articular chondrocytes, thus driving their differentiation to mature chondrocytes [58]. According to our results, the presence of angular chondrocyte-like cells expressing *Sox9* and *Col2a1* increases primarily in the BDR region of the IpL osteoligamentous junction at 5dpp and in hyaline cartilage at 10dpp. At this time, the systemic and constant low levels of oestrogen may be sensed by osteochondral progenitor cells and differentiated chondrocytes at the pubic junction. Thus, oestrogen/receptor signalling can lead to *Sox9* and *Col2a1* expression, thereby favouring the differentiation of progenitors in mature chondrocytes and the proliferation of chondrocytes at cartilaginous pads as is necessary for PS recovery at 10dpp.

Therefore, our results demonstrate that the groups of round osteochondral progenitor cells that test positive for Sox9, Col2a1, Dcx and Runx2 that are located next to subchondral bones at NP PS may be influenced by both hormonal levels and compressive forces during pregnancy and postpartum. Similar to the situation observed during the normal healing of bone fractures and in human articular cartilage at late stages of osteoarthritis [50, 51, 59], these osteochondral progenitors can proliferate, migrate and provide cells that will eventually differentiate into chondrocytes to promote tissue recovery at the interpubic joint.

Finally, our results showed that at the end of pregnancy and at 5dpp during IpL remodelling, *Dcx* was expressed by either elongated or round osteochondral progenitors at the BPR regions of the IpL osteoligamentous junction, where *Sox9* is also expressed at D19 and 5dpp. Cells with similar characteristics are found during limb formation in embryonic cells that direct cartilaginous framework shaping and osteochondral and articular cartilaginous tissue formation [47, 48]. However, the decrease in *Dcx* expression in PS 10dpp hyaline cartilage tissue detected in this work indicates that chondrocytes present in this tissue are fully committed to the endochondral lineage, since chondrocytes with markedly decreased *Dcx* expression are involved exclusively in endochondral ossification and bone growth plate formation in adult mice [41, 47, 48]. Therefore, we believe that *Dcx* expression and immunolocalization are related mainly to PS osteochondral progenitor chondrocytic lineage commitment during postpartum recovery.

In summary, the results of this study showed that the subchondral region of the symphyseal pubic bones and the BPR of the IpL osteoligamentous junction directly supply osteochondral progenitors for C57BL/6 mouse PS remodelling during pregnancy and postpartum, as demonstrated by the morphological analysis and expression profiles of relevant molecular markers. Additionally, this process might recapitulate embryologic chondrocytic differentiation features that modulate *Sox9*, *Col2a1*, *Dcx and Runx2* expression and facilitate the successful recovery of hyaline cartilaginous pads after 10dpp. The results of the morphological and molecular analyses clarified PS remodelling, which was shown to represent a valuable proof-of-concept model that can be used to investigate the mechanisms involved in cartilage restoration in adult animals.

## Materials and methods

### Animals

Virgin female C57BL/6/JUnib mice (three months old) were obtained from the Multidisciplinary Centre for Biological Investigation (CEMIB) of the State University of Campinas (UNICAMP). Mice were housed at 25 + 2°C under a 12-hour light/dark cycle with free access to water and standard pelleted rodent chow. Mating was encouraged by placing young females in cages with breeding males overnight. The presence of a vaginal plug indicated day 1 of pregnancy (D1). The animals were anaesthetized by intraperitoneal injection of xylazine chloride (100–200 mg/kg) and ketamine (5–16 mg/kg) (Agribrands do Brasil, Paulinia, Sao Paulo, Brazil), which was administered between 11 pm and 12 pm. After euthanasia by cervical dislocation, PS or IpL were removed from mice for processing. PS or IpL were obtained from the following groups: NP young mice, D19, 1dpp, 3dpp, 5dpp and 10dpp. Three animals comprised the experimental groups for analysis using light microscopy assays, and five animals comprised the groups used in the *in situ* hybridization, immunohistochemistry and immunofluorescence experiments, for a total of 126 animals. The animal experiments were conducted in accordance with the Guide for the Care and Use of Laboratory Animals issued by the National Institutes of Health (Bethesda, MD). All protocols using mice were approved by the Institutional Committee for Ethics in Animal Research (Comissão de Ética no Uso de Animais-CEUA/IB/UNICAMP, protocols 2430–1 and 3789–1). Mouse embryos at 14.5 days post coitum (dpc) were kindly provided by UNICAMP Gene Expression Laboratory (LARGE) (CEUA/IB/UNICAMP, protocol 3019–1).

### Light microscopy

PS and IpL were fixed with 4% paraformaldehyde (Merck, Darmstadt, Germany) in 0.1 M phosphate-buffered saline (PBS; pH 7.4) for 24 hours at 4°C. Decalcification was performed during five days in 5% ethylenediaminetetraacetic acid (EDTA, Mallinckrodt Baker, Phillipsburg, NJ, USA) and 2% paraformaldehyde in 0.1 M PBS, pH 7.4 for five days at 4°C. Tissues of three animals per experimental group (a total of 18 animals) were dehydrated in graded concentrations of alcohol, embedded in Historesin (Leica Microsystems, Heidelberg, Germany) and sectioned (3 μm) for staining with Giemsa [14]. Alternatively, interpubic tissues of an additional three animals per group (a total of 18 animals) were decalcified and dehydrated in graded concentrations of alcohol, embedded in paraffin, sectioned (5 μm), stained with Sirius Red F3B and observed with the aid of polarization microscopy to gain insight into the time-dependent changes of the microstructure of collagen fibres in the pubic joint during pregnancy [12]. Sections were examined and imaged under a Nikon Eclipse E800 light microscope (Nikon Corporation, Tokyo, Japan).

### *In situ* hybridization (ISH)

For gene expression analysis, mouse embryos at 14.5 dpc and interpubic tissues from five animals per study group (a total of 30 animals) were embedded in paraffin and serially sectioned (6 μm). Three slices of interpubic tissues representing each day of the study were placed on the same slide to optimize the ISH assay. The protocol used was adapted from Sensiate et al. [60]. Briefly, paraffin sections were treated with proteinase K (10 μg/ml) for 4 min at 37°C and then incubated in prehybridization solution at 57°C for 2 hours. Hybridization and post-hybridization temperatures were optimized for each gene of interest (Table 1). Antisense RNA probes for *Col2a1*, *Dcx*, *Sox9*, and *Runx2* were prepared according to Sensiate et al. [60]. Staining specificity was monitored using sense RNA probes. Sections were photographed under a Nikon Eclipse E800 light microscope.

**Table 1:** ISH probes: Primers, amplicon sizes and temperatures used in the hybridization assays. Gene-specific forward (F) and reverse (R) primers were used to generate antisense RNA probes for use in the in situ hybridization (ISH) assays. All R primers also contained the T7 RNA promoter sequence (***T7PS***) at the 5’-end (5´-TAATACGACTCACTATAGGGAGA-3´). The amplicon sizes and temperatures used in the ISH assays are also shown.

*Gene*	*Primers*		*Amplicon size (bp)*	*Temperatures*	Reference sequence (NCBI)
*Col2a1*	*F*	5’-TGGTGACAAGGGAGAAAAGG-3’	764	56°C	NM_001113515.2
	*R*	5’- ***T7PS*** AACCTTGAGCACCTTCAGGA-3’			
*Dcx*	*F*	5’-GGGGATTGTGTACGCTGTTT-3’	779	57°C	AF045547.1
	*R*	5’- ***T7PS*** TTGAGAGCTGACTGCTGGAA-3’			
*Runx2*	*F*	5’-CACTGCCACCTCTGACTTCT-3’	*752*	68°C	NM_001146038.2
	*R*	5’- ***T7PS*** CCTTGGTAAAGGGGACATCT-3’			
*Sox9*	*F*	5’-AGAGCGAGGAAGATAAGTTC-3’	454	58°C	NM_011448.4
	*R*	5’- ***T7PS*** ATTAGGAGAGATGTGAGTCT-3’			

### Immunohistochemistry (IHC) and immunofluorescence (IF) laser confocal scanning microscopy analysis

SOX9, COL2A1 and DCX protein localization was determined by IHC. Mouse embryos at 14.5 dpc were used as positive controls in all assays. Five samples of each experimental group (a total of 30 animals) were fixed as previously described without EDTA treatment, embedded in paraffin and transversely sectioned. After paraffin removal, IHC assays were carried out using an N-Histofine^(R)^ Simple Stain Mouse Kit (Nichirei Biosciences, Inc., Japan) with the following primary antibodies: SOX9 (1:50, SC 20095; Santa Cruz Biotechnology, Inc., California, USA), COL2A1 (1:200, LS C118414; Lifespan Biosciences, Inc., Seattle, Washington, USA), and DCX (1:200, SC-8066; Santa Cruz Biotechnology, Inc., California, USA). Sections were observed and photographed under a Nikon Eclipse E800 light microscope.

RUNX2 and chondroitin sulphate IF were performed in cryosections (8 μm) of PS, and IpL from five animals representing each day of the study (a total of 30 animals) was frozen in n-hexane that had been cooled in liquid nitrogen. Positive controls for RUNX2 and chondroitin sulphate IF reactions were, respectively, 8-μm sagittal cryosections of 14.5-dpc mouse embryos and 8-μm cryosections of NP PS. After sectioning, the slides were immersed in acetone at -20°C for 3 min and then washed in 0.1 M PBS, pH 7.4. After blocking with 1% bovine serum albumin for 30 min, the sections were incubated with RUNX2 (1:100, HPA022040; Sigma Life Science, USA) or chondroitin sulphate (1:300; BS-4800R, Bioss antibodies, Massachusetts, USA) primary antibody at -4°C overnight. The slides were then incubated with fluorophore-conjugated secondary antibodies (AlexaFluor 488 at 1:600; Abcam, Cambridge, UK). Nuclei were stained using 4,6-diamidino-2-phenylindole (SC-3598; Santa Cruz Biotechnology, Inc., California, USA). The slides were mounted using Fluoroshield mounting medium (ab 1041135; Abcam, Cambridge, UK) and visualized at the National Institute of Science and Technology on Photonics Applied to Cell Biology (INFABIC) instrument at the State University of Campinas using a Zeiss LSM 780-NLO confocal mounted on an Axio Observer Z.1 microscope (Carl Zeiss AG, Germany) with 20x and 40x objectives.

### Statistical analysis

Metachromatic hyaline cartilage tissue volumes in NP and at 10dpp were morphometrically estimated by point counting according to Foldager et al. [61], and the differences between numerical data obtained in both groups were analysed by performing a Mann-Whitney nonparametric test; differences with p<0.05 were considered significant, and the analysis was performed using GraphPad Prism 5.0 (GraphPad Software, Inc., California, USA). Differences in metachromatic hyaline cartilage tissue volumes between NP and 10dpp PS were obtained using the Mann-Whitney nonparametric test and plotted on a graph, and the mean SEM values are represented using bars.

## Supporting information

S1 Fig Expression of Sox9, Col2a1, Dcx and Runx2 at 14.5 dpc in rib and digit cartilage blastema. (A, B, E and F) Sox9 and Col2a1 mRNA and protein were localized to the inner cells of the rib cartilage blastema (arrows). (C and G) Dcx mRNA was expressed in digit blastema articular site cells (arrow), and protein was localized to rib cartilage blastema inner cells (arrow) and the perichondrium (D and H). Both Runx2 mRNA and protein were localized at the perichondrium of the rib cartilage blastema (arrow) and to mesenchymal and epithelial cells (1:2000 anti-DIG pod/H-O). Immunohistochemistry (IHC); Immunofluorescence (IF); *In situ* hybridization (ISH) experiments (A-G) Scale bars = 20 μm. (H) Scale bar = 50 μm.
(TIF)
