# Supplementary figures and images for: Time-dependent regulation of morphological changes and cartilage differentiation markers in the mouse pubic symphysis during pregnancy and postpartum recovery
Source: PLoS One. 2018 Apr 5;13(4):e0195304. doi: 10.1371/journal.pone.0195304 (PMC5886480; doi:10.1371/journal.pone.0195304)

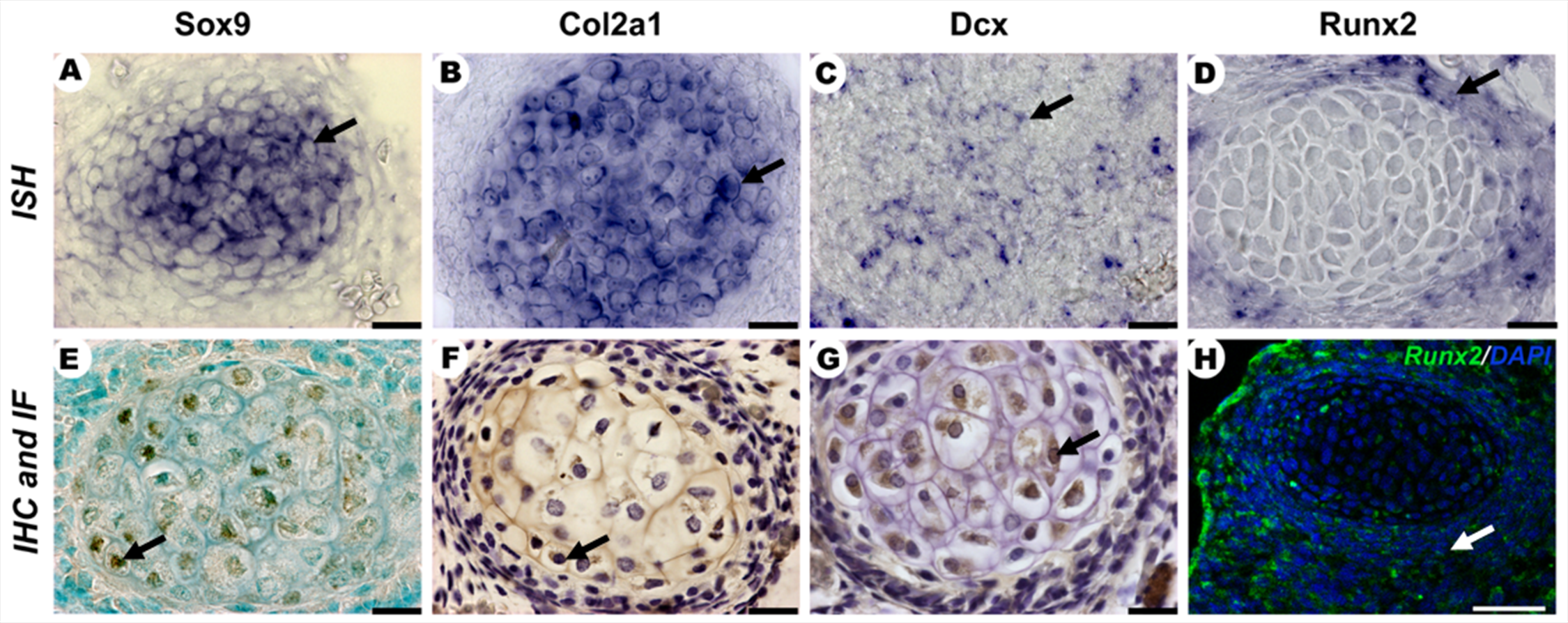

Supplement: S1 Fig — (A, B, E and F) Sox9 and Col2a1 mRNA and protein were localized to the inner cells of the rib cartilage blastema (arrows). (C and G) Dcx mRNA was expressed in digit blastema articular site cells (arrow), and protein was localized to rib cartilage blastema inner cells (arrow) and the perichondrium (D and H). Both Runx2 mRNA and protein were localized at the perichondrium of the rib cartilage blastema (arrow) and to mesenchymal and epithelial cells (1:2000 anti-DIG pod/H-O). Immunohistochemistry (IHC); Immunofluorescence (IF); In situ hybridization (ISH) experiments (A-G) Scale bars = 20 μm. (H) Scale bar = 50 μm. (TIF) [file pone.0195304.s001.tif]
